# Macroalgae Decrease Growth and Alter Microbial Community Structure of the Reef-Building Coral, *Porites astreoides*

**DOI:** 10.1371/journal.pone.0044246

**Published:** 2012-09-05

**Authors:** Rebecca Vega Thurber, Deron E. Burkepile, Adrienne M. S. Correa, Andrew R. Thurber, Andrew A. Shantz, Rory Welsh, Catharine Pritchard, Stephanie Rosales

**Affiliations:** 1 Florida International University, Deptartment of Biological Sciences, North Miami, Florida, United States of America; 2 Oregon State University, Deptartment of Microbiology, Corvallis, Oregon, United States of America; 3 Oregon State University, College of Earth, Ocean and Atmospheric Sciences, Corvallis, Oregon, United States of America; 4 Oregon Institute of Marine Biology, Charleston, Oregon, United States of America; King Abdullah University of Science and Technology, Saudi Arabia

## Abstract

With the continued and unprecedented decline of coral reefs worldwide, evaluating the factors that contribute to coral demise is of critical importance. As coral cover declines, macroalgae are becoming more common on tropical reefs. Interactions between these macroalgae and corals may alter the coral microbiome, which is thought to play an important role in colony health and survival. Together, such changes in benthic macroalgae and in the coral microbiome may result in a feedback mechanism that contributes to additional coral cover loss. To determine if macroalgae alter the coral microbiome, we conducted a field-based experiment in which the coral *Porites astreoides* was placed in competition with five species of macroalgae. Macroalgal contact increased variance in the coral-associated microbial community, and two algal species significantly altered microbial community composition. All macroalgae caused the disappearance of a γ-proteobacterium previously hypothesized to be an important mutualist of *P. astreoides*. Macroalgal contact also triggered: 1) increases or 2) decreases in microbial taxa already present in corals, 3) establishment of new taxa to the coral microbiome, and 4) vectoring and growth of microbial taxa from the macroalgae to the coral. Furthermore, macroalgal competition decreased coral growth rates by an average of 36.8%. Overall, this study found that competition between corals and certain species of macroalgae leads to an altered coral microbiome, providing a potential mechanism by which macroalgae-coral interactions reduce coral health and lead to coral loss on impacted reefs.

## Introduction

Corals typically host species-specific communities of bacteria [1] that perform a wide variety of context-dependent roles on reefs [2]. For example, some bacteria may ward off pathogenic microbes by occupying available physical niches on coral colonies and/or producing antibiotics [3], [4], [5]. Within coral skeletons, cyanobacteria and other endolithic organisms may be important nitrogen fixers [6], [7], [8] supplying 50–200% of their host’s nitrogen requirement [9]. Cyanobacterial symbionts may be especially important for providing nutrients to the host during stressful conditions, such as bleaching events, when other symbionts (e.g., *Symbiodinium* dinoflagellates) are not performing adequately [10]. Common coral-associated bacteria also may play a variety of other roles such as digesting recalcitrant carbon sources (e.g., chitin, cellulose), scavenging micronutrients (e.g., iron), and impacting elemental cycling [3], [11], [12]. Therefore, coral-associated microbes are likely critical for the maintenance of coral colony health and survival [4], [13].

Anthropogenic impacts such as climate change, eutrophication, and overfishing are important drivers of the loss of coral cover and biodiversity [14], [15]. These stressors appear particularly severe on reefs in the Caribbean Sea where corals have declined ∼80% in recent decades [16], [17], [18]. Concomitantly, the combined forcing of reduced herbivore abundance from overfishing, increased nutrient input, and loss of coral cover due to bleaching and disease has led to a significant increase in macroalgal cover on many Caribbean reefs [19], [20], [21]. Abundant macroalgae may reinforce a coral-depauperate state by facilitating the spread of coral diseases [22], [23], reducing the survival and growth of adult corals [24], [25], [26], [27], and/or preventing the recruitment of juvenile corals [24], [28], [29].

Despite evidence showing that increased macroalgal abundance has negative effects on corals, we understand little about the mechanisms by which macroalgal competition may impact the coral-microbial mutualism and how these impacts relate to overall coral fitness. Increases in macroalgal abundance may alter the normal microbial communities on corals and potentially trigger episodes of microbial disease [22], [23], [30], [31], [32]. Although it is unlikely that macroalgae mediate all coral disease outbreaks, shifts in macroalgal diversity and abundance likely influence the taxonomic and metabolic diversity of coral-associated bacteria. Shifts in the microbial community on corals may result in a decreased abundance of beneficial bacteria, which could potentially increase colony vulnerability to water-borne pathogens, thermal bleaching, or other stressors. Alternatively, the presence of macroalgal competitors may influence the growth of rare members of the coral microbiome or even directly vector new, harmful microbial taxa onto corals. Here, we test whether coral-macroalgal interactions affect the microbial community on the scleractinian coral, *Porites astreoides*, and whether such alterations impact coral health.

## Materials and Methods

### Study Site and Experimental Design

The Florida Keys Reef Tract consists of a large bank reef system located approximately 8 km offshore of the Florida Keys, USA, paralleling the island chain. Our study site, Pickles Reef (25° 00′ 05″N, 80° 24′ 55″W), is a 5–6 m deep relict spur and groove reef system located off Key Largo, FL. Live coral cover ranges between 5–10% and macroalgal cover often varies between 20–30% [30], [31].

In July-September 2009, coral-algal competition experiments were conducted over 10 weeks using the coral *P. astreoides* and the macroalgal species: *Dictyota menstrualis*, *Galaxuara obtusata*, *Lobophora variegata*, *Halimeda tuna*, and *Sargassum polyceratium. Porites astreoides* is an encrusting/mounding coral and is now a spatial dominant after recent declines of other corals such as *Acropora cervicornis* and *Montastraea faveolata* [32]. *Dictyota* spp., *H. tuna*, and *L. variegata* are often the most abundant components of the macroalgal community on reefs in the Florida Keys and often compete with corals [31]. All of the macroalgal species that were used tend to increase when herbivory rates decline and thus represent species that might compete with corals where overfishing is common [26], [27]. *Porites astreoides* colonies were collected from a nearby shallow inshore reef at 3 m depth. Experimental corals were fragmented, transported to our field site, and then common garden acclimated at 5 m on site for 2 weeks prior to the experiment. Algae were collected either from our field site or nearby Pickles Reef or Conch Reef. Experiments were conducted using cinderblocks (10×20×40 cm) as competition arenas. *Porites astreoides* fragments (∼15 cm^2^ surface area as determine with the tin foil method [33]) were attached to plastic mesh using marine epoxy. For each replicate experiment, a single *P. astreoides* colony was split to provide all the fragments that underwent manipulation. This was done to allow comparison in growth rates of *P. astreoides* among the different replicates without being biased by intraspecific growth rates. A total of six colonies were split providing six independent replicates and a ‘blocked’ experimental design. The mesh attached to each fragment was used to anchor the fragment to cinderblocks using cable ties with a single coral fragment on either end of a single cinderblock (Figure S1A). Approximately equal volumes (5 ml) of one of the five algal species were attached to the mesh next to each fragment, so that it was in direct contact with the coral (Figure S1A). One replicate of the experiment included each of the five macroalgal species plus a control randomly assigned to one of the three cinderblocks (Figure S1B). Controls were a treatment where no macroalgae were transplanted next to the coral. Each coral-algal pairing (including control) was replicated 6 times. The combination of macroalgal treatments on a specific cinderblock was randomized within each block of the experiment. Algae and cable ties were replaced every 1–2 weeks to minimize fouling by other algae or invertebrates. An exclosure of plastic mesh (2.5 cm diameter) was constructed around each cinderblock to prevent corallivores from preying on corals and herbivores from grazing on macroalgae.

To measure growth, corals were buoyant weighed at the beginning and end of the experiment [34]. Growth rates were calculated as g/cm^2^ of colony area/day. Differences in growth over the course of the experiment were assessed by comparing growth of each coral fragment in the macroalgal competition treatments to growth of the control within each experimental block via paired t-tests. Since data from the control corals were used for multiple statistical tests, we controlled for Type I errors using the Bonferroni-Holm correction [35]. This approach is analogous to applying a Dunnet’s post-hoc test to an ANOVA model, however, it allows the maximum amount of power from the paired experimental design as it takes into account the different intrinsic growth rates for each replicate colony that was divided into the treatment fragments. The Dunnet’s post-hoc test is based on repetitive t-tests that have been corrected for Type I errors [36], exactly as we have done here except we have used a paired t-tests. During the experiment, a storm dislodged several cinderblocks from the experiment and thus potentially compromised the health of these corals. However, none of the control fragments were damaged. In cases where a treatment was lost, the control from that replicate was not included in the statistical analyses, which lead to different replication for each treatment. Final replication for each algal treatment was: (1) control: n = 6, (2) *D. menstrualis*: n = 5, (3) *L. variegata*: n = 5, (4) *H. tuna*: n = 5, (5) *G. obtusata*: n = 4, and (6) *S. polyceratium*: n = 5.

### Isolation of Coral- and Macroalgal-associated Microbial Communities

To characterize the microbial community on each macroalgal species, small portions of each macroalgae (n = 5 of each species) were sampled from the coral-algal competition arenas prior to the experiment. A 1 cm^2^ portion of each macroalgal thallus was patted dry and swabbed with a sterile cotton swab to collect surface-associated microbes and avoid seawater-associated microbes. Swabs were placed in 15 ml conical tubes containing 10 mls of 95% ethanol and stored at 4°C. For microbial DNA extractions, swabs were placed in an o-ring sealed, 2 ml centrifuge tube containing 1.5 ml of lysis buffer (0.36 M NaCl, 45 mM EDTA, 1% SDS), vortexed for 15 minutes in a bead beater, and incubated at 65°C for 1.5 hours. DNA was archived and later extracted according to previously published methods [37].

At the conclusion of the experiment, corals were placed in sterile Whirl-paks, brought to the surface, and placed on ice. Once on shore, corals were rinsed in 0.2 µm filtered seawater to remove seawater associated microbes, weighed, separated from their mesh base, and placed in 50 ml conical tubes containing 30 ml of 95% ethanol and stored at 4°C (n = 4 per treatment). For microbial DNA isolation, coral tissue was removed using sterile razor blades, placed in 2 ml centrifuge tubes, and extracted using same method as described for the macroalgal swabs except that the coral samples were not bead beaten [37].

### Community Analyses of Coral- and Macroalgal-associated Microbes

Relative microbial taxonomic diversity was measured using terminal restriction fragment length polymorphisms (T-RFLP) [38], using the primer sets, FAM-Univ 9F and Univ 1509R, to amplify the 16S rRNA gene from each sample in two replicate 50 µl PCR reactions (10 µl of 5× buffer, 2.4 mM of MgCl, 0.2 µM each primer, 2.5 U of Taq polymerase, and 0.2 mM of each dNTP) using the following touchdown thermo-cycler program: 95°C 2 min, 34 cycles of 95°C 1 min, 55.6°C 1 min (−0.3°C), and 72°C 1 min, and a final extension of 72°C 5 min step [39]. Successful duplicate amplifications were combined and cleaned using the Promega PCR Wizard kit (Madison, WI). Seven samples (including both coral tissues and algae thalli specimens, notably the *G. obtusata* coralline algae) could not be amplified and were eliminated from the study. To normalize the amount of sample analyzed, exactly 960 ng of DNA from each pooled amplification was digested at 65°C for 4 h using the restriction enzymes Rsa1 and Hha1 from Promega (Madison, WI) and analyzed at Laragen, Inc (Culver City, CA) with a 0.5 µl GeneScan™ Liz1200 size standard and an ABI 3730 sequencer (Applied Biosystems). Overall this large dataset includes 84 TRFLP profiles (42 individual samples, and digested with two enzymes).

TRFs were determined using the Local Southern size-calling algorithm of the Peak Scanner Software Version 1.0 (Applied Biosystems). Sample versus TRFLP peak data matrices were constructed using a conservative threshold of 50 units above background. Peaks smaller than 50 base pairs (bp) and larger than 1200 bp (outside of the standard’s linear range) were removed *in silico*. Peak area was linearly related to peak height (r^2^ value >0.95), therefore, relative microbial taxa abundance data was obtained from peak heights following sample standardization including rounding to the nearest integer and a two basepair bin of fragment sizes [40]. Since the selected primers target a large portion of the 16S rRNA gene, TRFs can represent one or more bacterial and/or archaeal sequence fragments. Therefore, for the purposes of this paper, we refer to the bacteria and archaea as “microbial communities.”

One way analysis of variance (ANOVA) followed by Tukey post-hoc tests were used to identify if: 1) relative microbial abundance differed across macroalgae species and among the coral-algal competition experiment, 2) there were differences among specific microbial TRFs within each treatment, and 3) there were differences in diversity of the microbial taxa as a function of the treatment. The underlying assumptions of this test were determined graphically (homogeneity of variance) and using a Kolmogorov-Smirnov test (normality). A variety of transformations were applied (see results) when necessary to meet these assumptions. Diversity indices employed included species richness (total number of TFRLP peaks) and CHAO1 predicted relative taxonomic abundance [41].

Multidimensional analyses were used to identify whole microbial community differences among the macroalgae and treatments. These analyses, along with the later two measures of diversity listed above, were performed in PRIMER v. 6 [42]. Bray-Curtis similarity was used to compare log-transformed peak heights of microbial abundance and distribution data (as measured by TRFs). The similarity of these data was visualized using multidimensional scaling (MDS) plots. Significant differences between these microbial communities among the algal treatments were evaluated using an analysis of similarity (ANOSIM), and the identification of which taxa were most important at driving the differences among the groups was conducted using similarity percentage (SIMPER) analysis.

### Permit

Permit # FKNMS-2009-047 was obtained for this study from the Florida Keys National Marine Sanctuary.

## Results

### Corals Exposed to Macroalgae have Reduced Growth Rates

**Figure 1:**
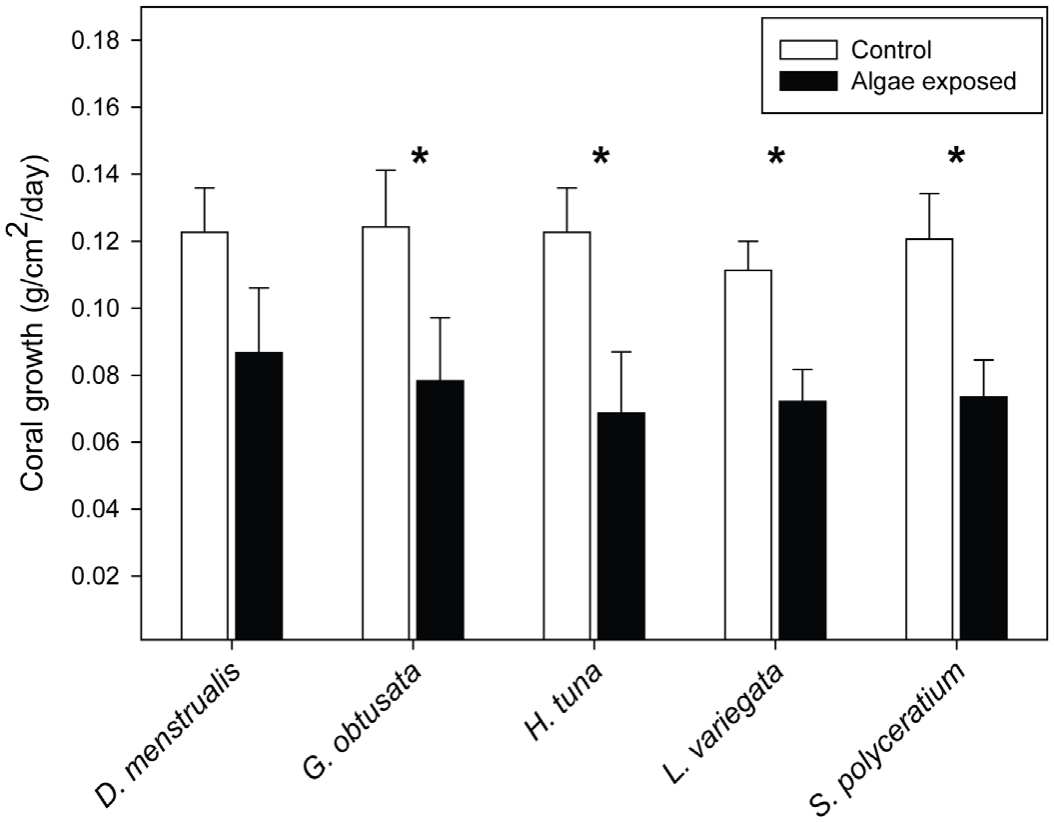
Effects of treatments on coral growth. Comparisons of growth rates (means ± SE) between corals competing with one of five macroalgal species vs. control corals. Each algal treatment in the block design had its own paired controls that had no algae. The number of controls were constant among treatments except when lost due to storm damage; in that instance the corresponding control was removed from the analysis. Statistics are from paired t-tests. *P*-values are based on Bonferroni-Holm correction for multiple comparisons with the controls. * *p*<0.05.

Macroalgal competition decreased coral growth rates by a mean of 36.8% (0.12±0.007 g cm^−2^ day^−1^ for control corals vs. 0.08±0.01 cm^−2^ day^−1^ across all corals with algal competitors; t = 3.4, P = 0.008). Yet, there were interspecific differences in how macroalgae affected coral growth. *Dictyota menstrualis* did not significantly suppress growth in *P. astreoides* (Figure 1). The other four macroalgal species all lowered coral growth rates relative to controls with no macroalgal competitors.

### Microbial Community Composition Differs Among Macroalgal Species and Among Control and Treated Corals

Among the macroalgae, 429 individual TRFs were identified while in the coral-algae experiments 232 TRFs were identified. Using the relative abundance of each TRF, mean microbial community profiles were generated for all the different macroalgae and coral-algae experimental specimens. A majority of these TRFs were singletons or comprised less than 3% of any one community profile. However, forty-one TRFs were identified that compromised a mean of 3% or more of any community profile (Figure S2). All samples demonstrated variation in the combination of these TRFs with the macroalgae samples only containing 5 TRFs (63, 421, 424, 826, 1048) in common with any coral community. The number of major TRFs in each sample varied from as low as 2 (*G. obtusata* thalli) to as many as 20 (*D. menstrualis* exposed corals). Overall, while there were more individual TRFs identified among macroalgal samples, there were only ∼9 major TRFs (mean ± SE: 9.40±2.0 TRFs) that comprised more than 3% of algae microbial community. In contrast, coral samples contained more than twice the number (mean ± SE: 16.8±1.30 TRF) of major TRFs.

**Figure 2:**
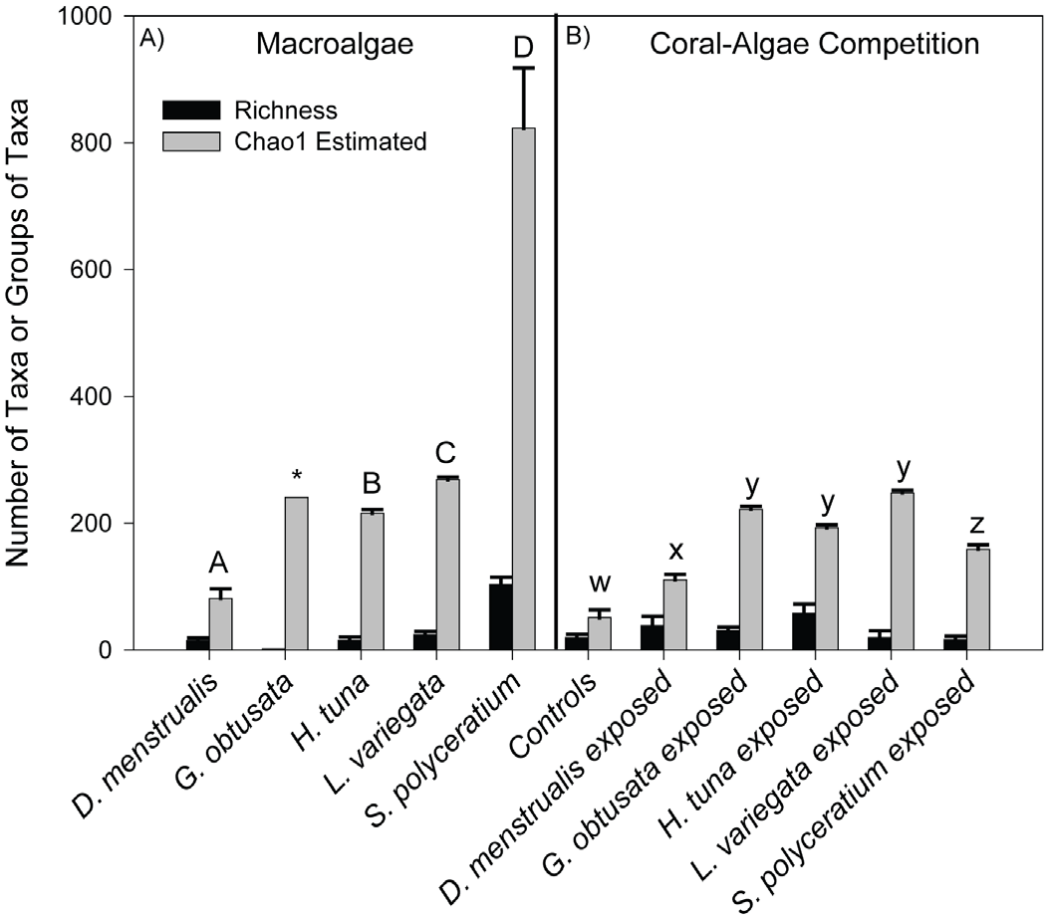
Comparisons of relative bacterial diversity among macroalgal species and among coral-algal competition treatments. The presence of individual TRFs were used to determine bacterial species richness (black bars) and CHAO 1 estimates (grey bars) were used to predict the relative number of taxa (means ± SE) in macroalgae (A) and on corals challenged with macroalgae (B). Letters represent significant differences (*p*<0.05) in CHAO1 estimates among sample types; richness was not found to be significantly different among samples. Differences among macroalgae are denoted by uppercase letters A-D while difference among coral-algae treatments and controls are denoted by lowercase letters w-z. *Galaxaura obtusata macroalgae data were not included in statistical analysis due to low replication.

**Figure 3:**
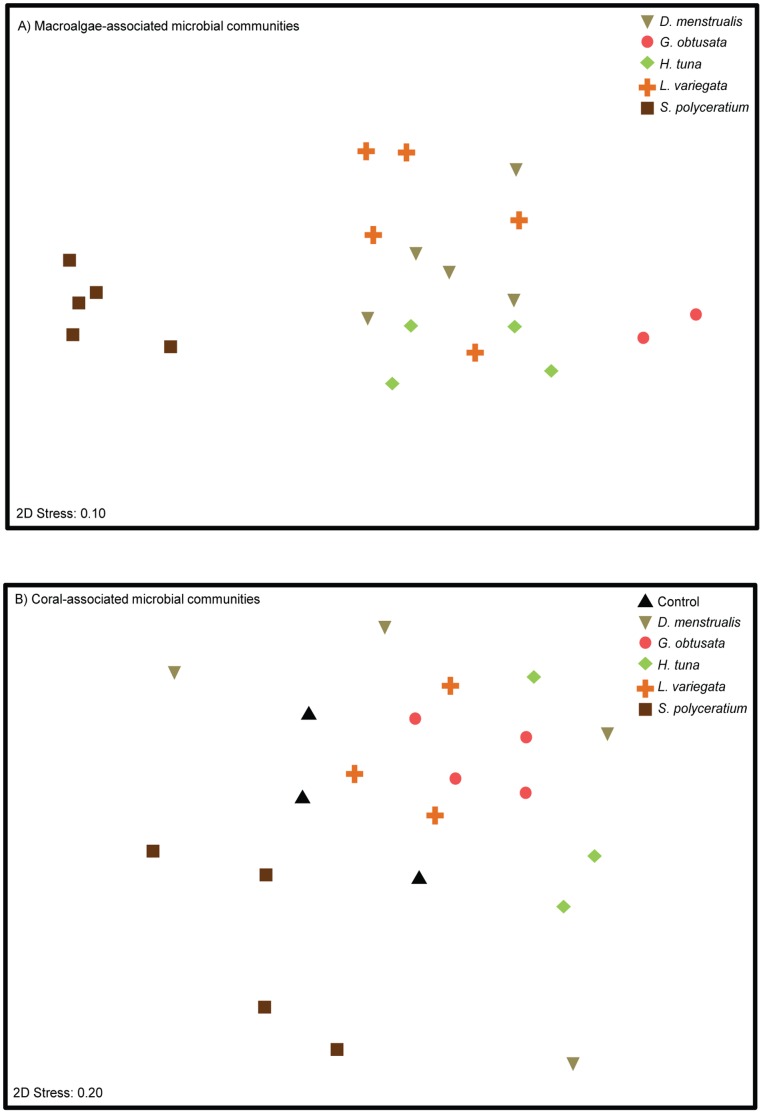
Microbial communities on algal thalli. Multidimensional ordination of Bray-Curtis similarity of microbial communities found on macroalgae (A) and on corals in competition with different macroalgal species (B).

Using all of the TRFs it was found that relative taxonomic richness of the microbial community on macroalgae varied ∼80 fold among species (Figure 2A). *Sargassum polyceratium* hosted the highest species richness (mean of 103 TRFs observed, black bars). CHAO 1 estimators (grey bars) also predicted that *S. polyceratium* thalli statistically contained the most species-rich microbial community (ANOVA F_3,15_ = 62.85, p≤0.001; post-hoc results indicated on Figure 2A) compared to all other macroalgae (mean ± SE: 822.0±9.26 taxa). Of the coral treatments, control corals contained statistically fewer taxa compared to all other macroalgal treatments, with an observed species richness of only 19 TRFs resulting in a mean of 51 (SE ±12.18) predicted microbial taxa or groups of taxa (ANOVA F_5,15_ = 88.0, p≤0.01; see Figure 3B for post-hoc results). Species evenness also was determined for all algae species and coral-algae interaction experiments, but no major differences among macroalgae or among coral treatments were detected.

To determine if microbial communities differed among macroalgal species and among corals in competition with macroalgae, multi-dimensional scaling (MDS) and analysis of similarity (ANOSIM) were performed. The microbial communities present on all macroalgae, except *G*. *obtusata*, were different from the communities present on the coral samples (Table S1). Further, distinct microbial communities were found associated with different macroalgal species, with the exception for *G. obtusata* (Figure 3A, circles) and *H. tuna* (diamonds), which were not significantly different from one another (Table S1). In an MDS plot containing just the macroalgal samples, *S. polyceratium*-associated microbial communities were particularly distinct, forming a tight cluster (squares; Figure 3A). The similarity of microbial communities within macroalgal samples of a given species ranged from a low of 32% for *L. variegata* to a high of 67% for *G. obtusata* (Table S2). The microbial community associated with *S. polyceratium* was highly (mean 92.2% ±1.93 SE) dissimilar from all other macroalgal species (Table S2). For example, TRF 895 was unique to *S. polyceratium* and contributed most to its grouping, yet this TRF still only comprised ∼7% of the community.

Community analysis also revealed that corals in competition with certain macroalgae harbored microbial communities significantly different from those on control corals (Figure 3B). Specifically, exposure to *S. polyceratium* (Figure 3B, squares) resulted in microbial communities that were different from those on control corals (triangles), as well as from microbial communities sampled on all other coral-macroalgal pairings (Table S1). Exposure to *G. obtusata* (circles) also significantly altered microbial communities relative to controls. Further, treated corals exhibited higher within-group community variation (mean 33.56% ±4.63 SE) than the control corals, which had the most similar (∼44%) microbial communities of all the samples (Table S3). Microbial communities on corals exposed to *D. menstrualis* showed the most variation within only 16% similarity to each other. On average control corals were 75% dissimilar to the corals exposed to macroalgae (mean 74.82% ±1.78 SE).

**Figure 4:**
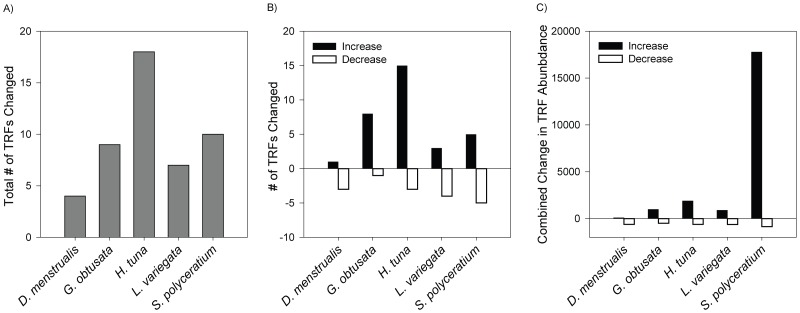
Significant Changes in Individual TRFs. Individual TRFs were compared between control corals and corals exposed to different macroalgal species. The relative total number of TRFs changed (A), the number of relative increases and decreases in individual TRFs (B), and the combined mean change in relative TRF abundance (C) compared to control TRFs.

Analysis of every TRF identified from the *P. astreoides* samples showed that a total of 15 TRFs were significantly different across the macroalgal treatments (Table S4). All macroalgal treatments produced at least one significant change in the abundance of a TRF as compared to TRFs associated with controls (Figure 4A). Corals exposed to *D. menstrualis* had the fewest TRF changes (i.e., two), while corals challenged with *H. tuna* had the most altered TRFs (i.e., 12). Observed TRF changes included significant increases and/or decreases, depending on the treatment (Figure 4B). For example, *S. polyceratium* exposure led to four TRF increases and two decreases, while *L. variegata* led to only a single TRF increase but three TRF decreases. Furthermore, the relative amount that each TRF changed compared to the control was different across treatments (Figure 4C). While all decreases in TRF abundance were similar across the treatments (from −75 to −146 units), the increase in TRF abundance in corals exposed to *S. polyceratium* (>12,000 units) was an order of magnitude greater than TRF increases in any of the other macroalgal treatments (≤2,000 units).

**Figure 5:**
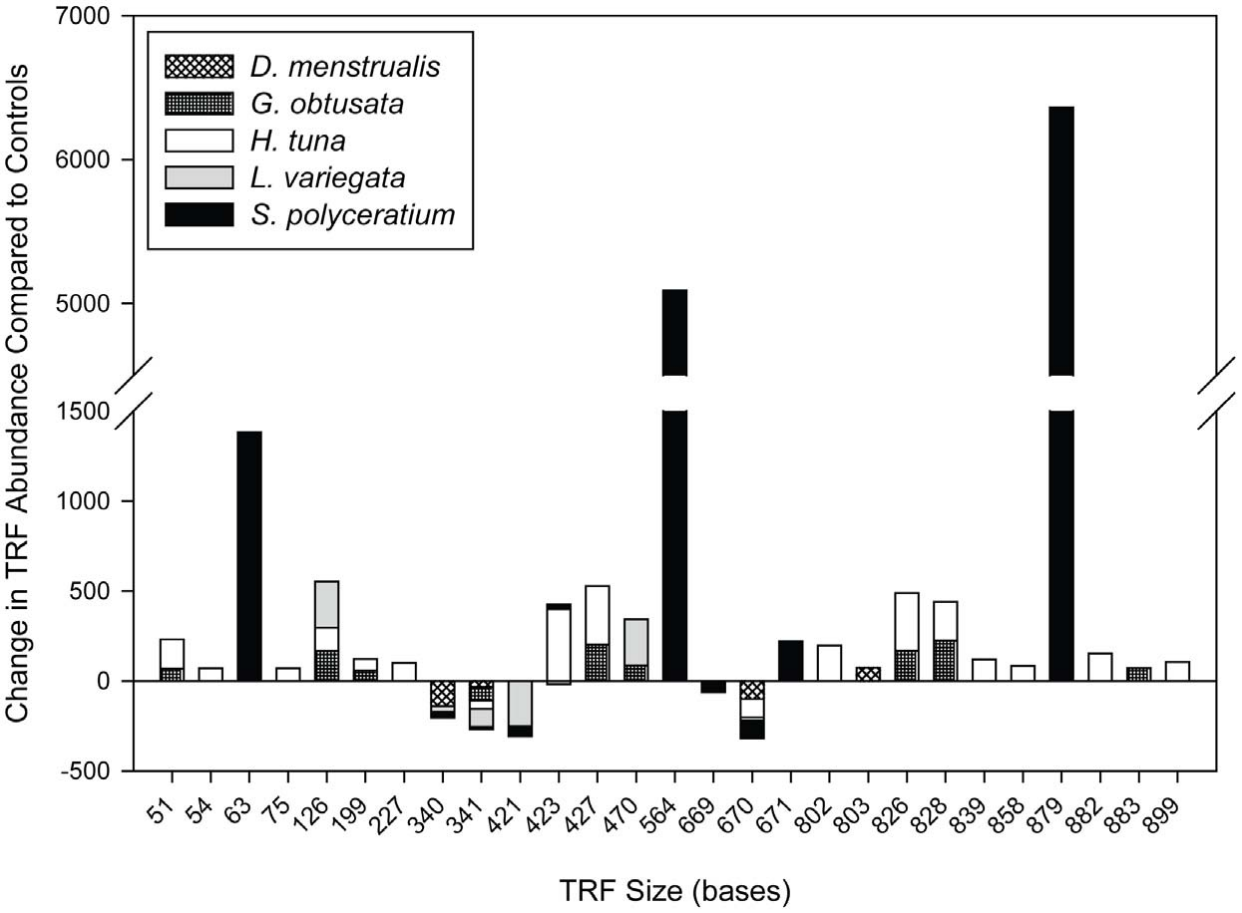
Contribution of Individual TRFs to Changes in Relative TRF Abundance. Changes in mean individual peak heights for specific TRFs for macroalgal competition treatments relative to control corals. Positive/negative values indicate an increase/decrease of a bacterial TRF on coral fragments in competition with a given macroalgal species, compared to TRFs on control corals.

Changes in the abundances of individual TRFs were complex (Figure 5); the abundance of some TRFs was altered by more than one macroalgal treatment while other TRFs changed within a single treatment only. For example, TRF 341 was reduced in every macroalgal competition treatment, whereas TRF 126 increased in response to *G. obtusata*, *H. tuna*, and *L. variegata*. *Halimeda tuna*-exposed corals had 6 unique TRF increases (TRFs 75, 227, 802, 858, 882, 899). TRFs 63, 564, and 879 were elevated only in corals exposed to *S. polyceratium*; increases in these TRFs were substantial relative to other observed TRF increases (Figure 5).

## Discussion

### Macroalgae Significantly Alter the Structure of Coral-Associated Microbial Communities

Our findings suggest that direct competition with some macroalgal species increases the taxonomic variability of microbes on *P. astreoides*. Similar decreases in the specificity of the coral microbiome have previously been observed in corals that differed in their health state and/or proximity to sewage outflows [39]. These increases in microbial community variability may be a general response to disturbance as has been seen in communities of macro-organisms [43]. Although the overall microbial community significantly changed only on corals in competition with two macroalgal species (*S. polyceratium* and *G. obtusata*) (Figure 3B; Table S1), some microbial taxa were significantly altered on all corals challenged with macroalgal competitors (Figure 5). Four types of alterations in the microbial TRFs associated with corals in competition with macroalgae were identified. First, some taxa present on control corals increased in abundance on macroalgae-treated corals. This increase was the least frequently observed change to microbial TRFs, occurring only with TRF 63. This TRF was present on every coral and macroalgae tested, and drove much of the clustering among microbial communities (Tables S2 & S3). Yet, the abundance of TRF 63 was significantly altered only on *S. polyceratium*-exposed corals, where it increased 4-fold relative to control corals (Figure 5).

Conversely, a bacterial taxon previously documented [3] to be a member of the *P. astreoides* holobiont always declined on corals in competition with macroalgae. TRF 341 was the most abundant TRF comprising ∼50% of the community in control corals, but its presence was reduced in every macroalgal treatment to below the detection level. Further, this TRF was not observed on any of the macroalgal species in this study. Rohwer et al. [3] first discovered TRF 341 on *P. astreoides* in Panama. TRF 341 was the most commonly identified taxa [3] (comprising an average of 61% of clones detected), and it was therefore named *P. astreoides* 1 (PA1). Sequencing identified PA1 as a γ-proteobacteria (GenBank accession # AF365457) [3]; the TRF 341 observed in corals in this study also represents the PA1 phylotype. PA1 has also previously been identified in *Diploria strigosa* corals as TRF 342 [39], where it exhibited lower abundances in diseased colonies, relative to apparently healthy conspecifics. Together, these previous studies have indicated that TRF 341 (PA1) is a likely important, but context dependent, mutualistic symbiont of corals. With the observation that there is concomitant reduction in PA1 abundance and coral growth in this study, it is possible that the loss of coral-associated microbial symbionts can potentially lead to reduced coral health. Alternatively the loss of this symbiont may have arisen from altered coral physiology that resulted from exposure to the algae. Both hypotheses should be tested in the future.

A third group of TRFs were present in control corals and changed non-uniformly across the macroalgal treatments. For example, TRF 423 increased in two of the competition treatments (*S. polyceratium* and *H. tuna*) but decreased in another (*L. variegata)*. These data suggest that competition with macroalgae can have idiosyncratic effects on coral-associated microbial communities depending on the species of macroalgal competitor and, potentially, the mechanisms that macroalgae use to compete with corals (e.g. allelopathy, abrasion, smothering).

The last and most commonly identified alteration to microbial communities was the detection of TRFs on macroalgal-treated corals that were not observed on controls. In our study, this included TRFs 75, 126, 227, 564, 802, 826, 828, 858, 879, and 899. We additionally searched for these TRFs within datasets generated from nine other *P. astreoides* colonies collected from this same reef in a long-term study (Burkepile & Vega Thurber, unpublished data), and TRFs 227, 564, 828, 858, 879, and 899 were never detected in these unmanipulated colonies (data not shown). This apparent relaxation of specificity in coral-microbial associations may represent colonization of corals by opportunist microbes during disturbance events.

The most significant taxon introduction to macroalgae-treated corals was TRF 879. This TRF was not detected from any control corals (n = 3) or local unmanipulated corals (n = 9) but represented up to ∼53% (mean 42.6% ±6.11 SE) of the microbial community on corals in competition with *S. polyceratium*. TRF 879 was not detected from thalli of *S. polyceratium*, and therefore does not appear to be vectored directly to the coral by the algae. In contrast, TRF 564 also was not observed on controls or local corals but was abundant on corals exposed to *S. polyceratium* (mean ∼26% of the coral-associated microbial community). Importantly, however, TRF 564 was detected from *S. polyceratium* thalli, albeit at lower relative abundances (∼1.5% of the algae-associated microbial community) than on treated corals, and TRF 564 was never detected on any other type of macroalgae. Taken together, these data indicate that TRF 564 was vectored from *S. polyceratium* to *P. astreoides* colonies and induced to proliferate. Although we currently do not know the identity of this TRF 564 microbial taxon, to our knowledge, this is first example of a macroalga vectoring a microbe to a coral. An alternative hypothesis is that TRF 564 also is present in the overlying water column and thus the surrounding seawater could have contributed to the increase in the relative abundance of this TRF on corals after exposure to the *S. polyceratium* algae. However, if that were the case, then TRF 564 should have been present in all of the samples (like TRF 63), yet, it was only ever detected on thalli of the *S. polyceritium* algae and corals exposed to that same algae. Therefore it is more parsimonious to suggest that TRF 564 is vectored from the algae to the corals.

While these data clearly demonstrate alterations in microbial diversity on corals, a caveat of TRFLP analysis is that it is not as sensitive at detecting rare members of the community, compared to other methods such as pyrosequencing [44]. It cannot be ruled out that rare members of the coral and macroalgal microbiomes were not detected using this technique. Therefore, it is possible that some microbial taxa, which appeared to be present on macroalgae-treated corals but not on controls, were in fact also present on control corals but below the detection threshold of our TRFLP analysis. For example, TRF 564 could normally be a rare member of the coral microbiome, whose growth is highly stimulated by the presence of *S. polyceratium*. Nevertheless, such dramatically large shifts in any one member of the microbiome in response to algal competition (e.g., from undetectable to 30% of the community) are likely to affect the metabolism of the coral holobiont [45], [46] with potentially adverse consequences for the coral.

Yet, our analysis did indicate that minor members of the coral microbiome were affected by interactions with macroalgae. While we found that the major (≥3% of any one community) microbial members of coral and algae were different among species and coral treatments, only 60% of the TRFs that were significantly altered on corals exposed to algae were from this majority. Rare taxa were also significantly altered, and contributed to 40% of all the significant individual TRF changes. These rare TRFs that were altered included: 51, 54, 199, 227, 802, 803, 839, 858, 882, 883, and 889. Together these data suggest that members of both the coral’s common and rare biosphere are impacted by algal interactions.

### Mechanisms Driving Macroalgal-Induced Changes to the Coral Microbiome

Several mechanisms could drive shifts in microbial abundance and community structure on corals competing with macroalgae. Exudates and surface bound compounds, including organic carbon and allelopathic chemicals may provide the mechanism that resulted in reduced coral growth and microbial community shifts in corals in contact with macroalgae. One perturbation that shifts microbial communities is an increase in the available food for heterotrophic bacteria, including those that feed upon dissolved organic carbon (DOC) [47], [48]. Some macroalgae exude DOC into the surrounding water column and may stimulate microbial growth at the expense of coral health [22], [49]. These mechanisms may be species-specific to different macroalgae or could differ according to morphology or growth rates. Patterns of DOC release vary widely among algal species and are influenced by algal growth form and morphology [50], [51]. Therefore, the differential DOC released by each of these macroalgal taxa may drive the species-specific shifts of the microbial community as well as the changes in coral growth that we observed. In one recent study, *Sargassum dentifolium* had the highest DOC exudation rates among nine species of benthic macroalgae [51]. If this is a common trend to the *Sargassum* genera, then DOC release may explain why corals exposed to *S. polyceratium* had the greatest shift in microbial community compared to all other treatments (Table S1).

In addition to DOC exudates, some macroalgae produce allelopathic surface compounds that directly alter the growth of surface bacteria on corals [52], [53]. Morrow et al. [53] found that of eight macroalgal species, *L. variegata* showed the most inhibitory effects against coral-associated microbial growth. Our data corroborate this effect, in that the competition between *L. variegata* and *P. astreoides* had the greatest inhibition of microbial taxa and also strongly depressed coral growth. In contrast, Morrow et al. [53] reported that *D. menstrualis* extracts had significant effects on coral-associated microbial dynamics, but in our study, this macroalga had the least impact on coral growth and coral-associated microbial communities. Likewise, *H. tuna* extracts had minimal effects (stimulatory or inhibitory) on coral-associated bacteria [53], yet in this study competition with *H. tuna* resulted in the most significant number of individual microbial taxa changes on corals (Figure 4). It could be that the changes in microbial growth documented by Morrow et al. [53] in short laboratory growth assays of several days were ephemeral and that these shifts do not persist under the more realistic field conditions of our experiment. Regardless, allelopathic interactions may be responsible for some shifts in microbial abundance in response to algal competition, but the role of allelopathy likely differs significantly among macroalgal species.

Hypoxia, vectored bacteria, and physical abrasion may also be important mechanisms that drive changes in the microbial communities on corals. When in contact with colonies, some macroalgae may create areas of persistent hypoxia, which facilitate changes in the coral microbial community [22], [54], [55]. Hypoxia is generated more commonly by filamentous turf algae or macroalgae that grow prostrate along a coral surface (e.g., *Dictyota* or *Lobophora*), rather than by upright macroalgae (e.g., *Sargassum* or *Halimeda*) [54]. Finally, macroalgae may be important vectors of bacteria that are not otherwise found on coral surfaces. As we show here, *S. polyceratium* introduced a microbe (i.e., TRF 564) to its coral competitor that was not observed in any other coral treatments or macroalgal species in this study. The microbial community on *S. polyceratium* appeared markedly different from other algal species in terms of taxonomic composition (Figure 3B). To our knowledge *S. polyceratium*-associated microbial communities have not previously been examined, yet, this macroalga is of particular interest as *Sargassum* spp. often increase on reefs when herbivores are removed [24], [26], [27], and the increase of *Sargassum* spp. on these reefs has led to lower coral recruitment, growth, and survivorship [24], [56].

One drawback of our experimental design is that we did not include an algal mimic treatment: (e.g., a piece of plastic placed in physical contact with coral fragments). Such a treatment would have indicated if the effect of live macroalgae on microbial communities and coral growth was a result of multiple factors or solely physical abrasion or shading. However, previous studies of coral-algal competition have shown that algal mimics have minimal effects on coral growth and health [52], [56]. Further, the fact that different algal species had different effects on coral growth, as well as coral-associated microbial communities, suggests that macroalgae impact corals in species-specific ways, rather than via general physical contact or shading.

Interactions with macroalgae could also lead to direct downstream changes in coral physiology (e.g., differential mucus layer polysaccharide composition and output); these physiological shifts could ultimately result in alterations to the coral microbiome. We suggest, however, that a direct or combined effect of macroalgae on the microbial community and coral host is most likely, given that past work has demonstrated that: (1) applications of DOC and alleopathic compounds to corals alter microbial growth rates *in situ* [48], [57], and (2) the application of antibiotics alleviates many of the negative effects of algae competition on corals [22].

### Conclusions

This study is among the first to empirically and quantitatively analyze shifts in coral-associated microbial communities resulting from competition with macroalgae. We show here that the presence of certain macroalgal species reduces the growth rate of the coral *P. astreoides*. These reduced coral growth rates occurred concomitantly with changes in their microbial community composition. Furthermore, contact with macroalgae can relax coral-microbial specificity, allowing microbial taxa that are not normally associated or exceedingly rare with a given coral host to become established. Given the increasing abundance of macroalgae in tropical coastal environments, interactions among macroalgae, corals, and microbes are likely to play a role in shaping the ecology of future reefs.

## Supporting Information

Figure S1 Schematic and Picture of Coral-algal Competition Experiment. (A). The combination of macroalgal treatments on a specific cinderblock (e.g., *D. menstrualis* and *H. tuna* on Cinderblock 1 below) was randomized within each block of the experiment. (B). The figure represents one complete block containing one replicate of each of five algal species treatments and the no-algae control. Fragments of *Porites astreoides* within a block of the experiment were all generated from the same original colony in order to minimize intraspecific differences in coral growth patterns.
(DOCX)

Figure S2 Pie Charts of Major TRFs in Each Coral Treatment of Algae Thalli as Measured by Mean Relative TRF abundance. TRF peak heights were averaged and percent contribution to the community measured. Any TRF that represented ≥3% of the community was plotted in the pie charts.
(DOCX)

Table S1 ANOSIM Results of Macroalgal-associated and Coral-associated Microbial Communities. Global R is 0.772 and significance level of sample statistic is 0.001. Bold text indicates a significant difference.
(DOCX)

Table S2 SIMPER Analysis of Macroalgae-associated Microbial Communities. Bold indicated total percent similarity. The most similar (Sim) or dissimilar (Diss) TRFs are followed by their average contribution to similarity or dissimilarity between two the macroalgae taxa.
(DOCX)

Table S3 SIMPER Analysis of Coral-associated Communities After Prolonged Contact with Macroalgae. Bold indicated total similarity. The most similar (Sim) or dissimilar (Diss) TRFs are followed by their percent contribution to the total similarity or dissimilarity.
(DOCX)

Table S4 TRF ANOVA Data for Coral-Algal Competition Experiments – Statistical values for one way ANOVA on TRF abundance data. C = control corals, D = *D. menstrualis* exposed corals, G = *G. obtusata* exposed corals, H = *H. tuna* exposed corals, L =  *L. variegata* exposed corals, and S = *S. polyceratium* exposed corals.
(DOCX)
