# Supplementary figures and images for: Macroalgae Decrease Growth and Alter Microbial Community Structure of the Reef-Building Coral, Porites astreoides
Source: PLoS One. 2012 Sep 5;7(9):e44246. doi: 10.1371/journal.pone.0044246 (PMC3434190; doi:10.1371/journal.pone.0044246)

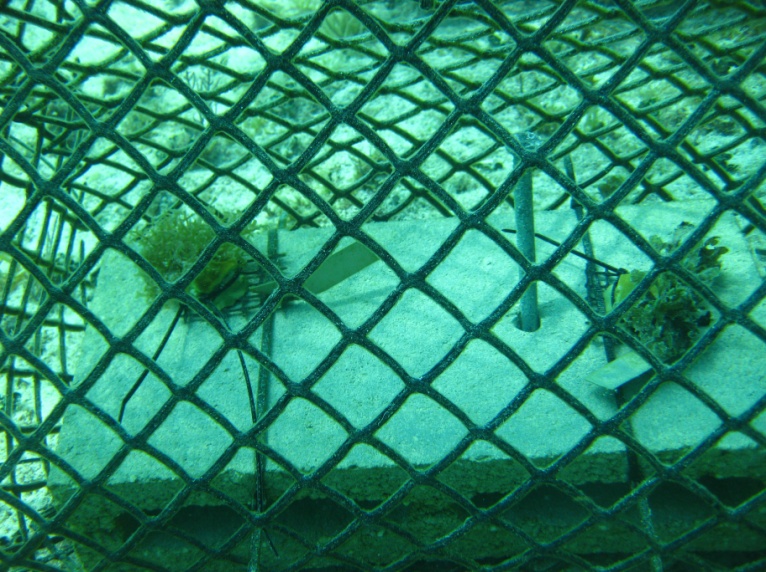
A)

B)


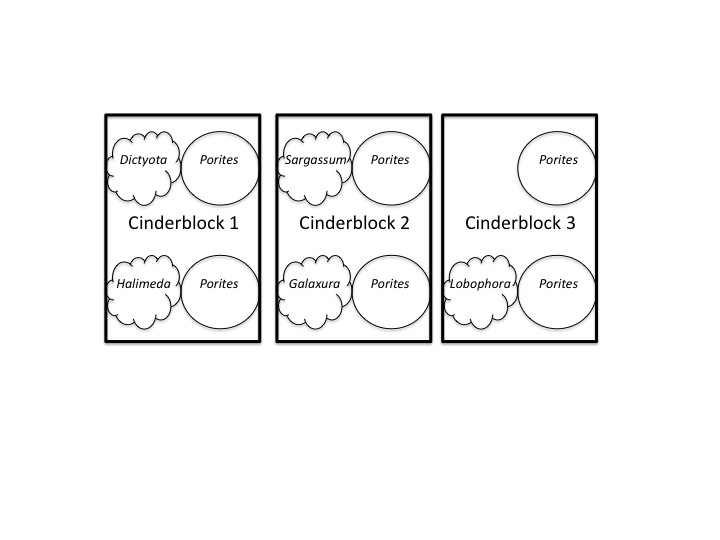

Supplement: Figure S1 — Schematic and Picture of Coral-algal Competition Experiment. (A). The combination of macroalgal treatments on a specific cinderblock (e.g., D. menstrualis and H. tuna on Cinderblock 1 below) was randomized within each block of the experiment. (B). The figure represents one complete block containing one replicate of each of five algal species treatments and the no-algae control. Fragments of Porites astreoides within a block of the experiment were all generated from the same original colony in order to minimize intraspecific differences in coral growth patterns. (DOCX) [file pone.0044246.s001.docx]

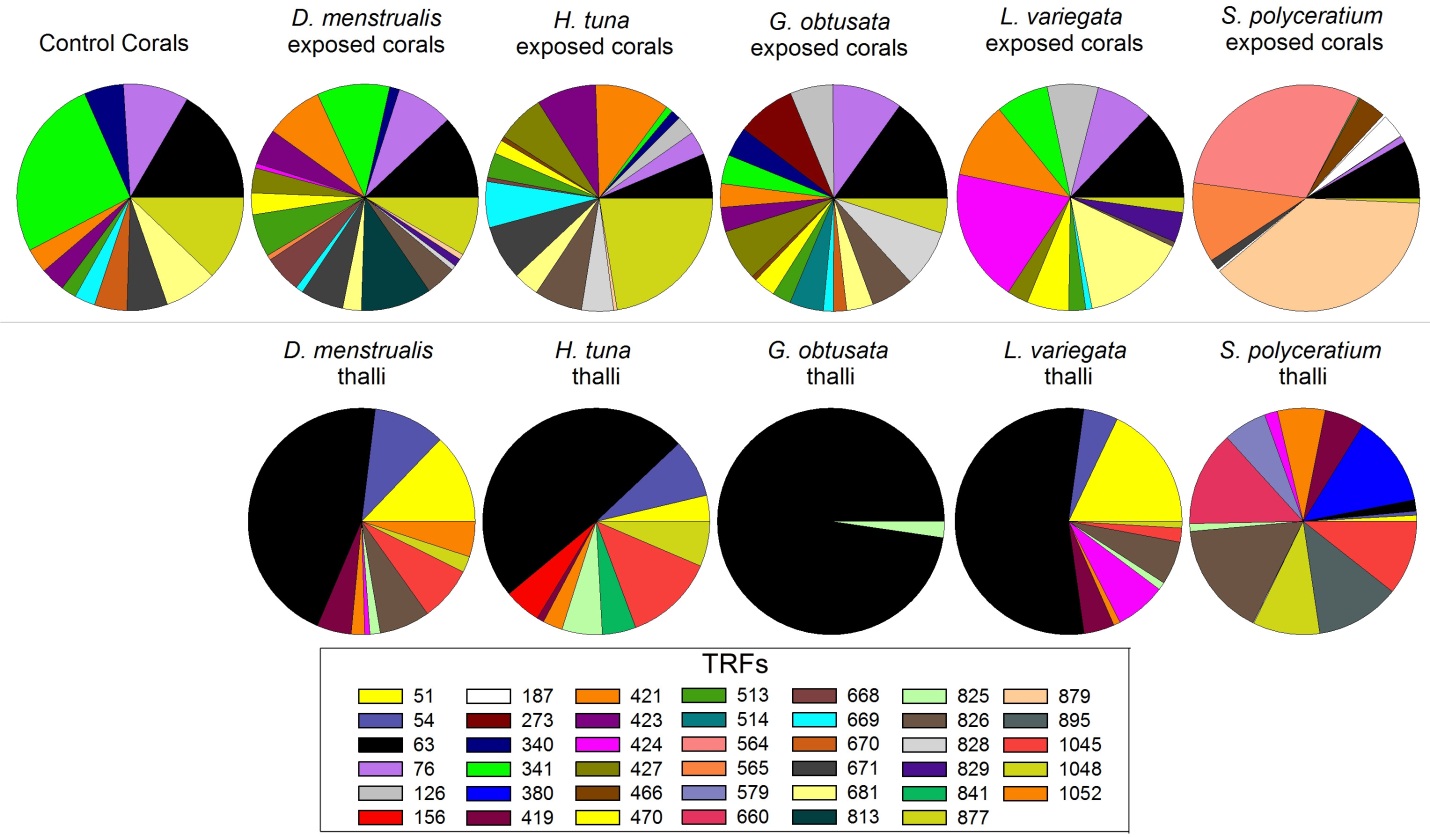

Supplement: Figure S2 — Pie Charts of Major TRFs in Each Coral Treatment of Algae Thalli as Measured by Mean Relative TRF abundance. TRF peak heights were averaged and percent contribution to the community measured. Any TRF that represented ≥3% of the community was plotted in the pie charts. (DOCX) [file pone.0044246.s002.docx]
