# Supplementary material for: Macroalgae Decrease Growth and Alter Microbial Community Structure of the Reef-Building Coral, Porites astreoides
Source: PLoS One. 2012 Sep 5;7(9):e44246. doi: 10.1371/journal.pone.0044246 (PMC3434190; doi:10.1371/journal.pone.0044246)
Supplement: Table S1 — ANOSIM Results of Macroalgal-associated and Coral-associated Microbial Communities. Global R is 0.772 and significance level of sample statistic is 0.001. Bold text indicates a significant difference. (DOCX) [file pone.0044246.s003.docx]

Supplemental Table 1. ANOSIM of Macroalgal-associated and Coral-associated Microbial Communities. Global R is 0.772 and significance level of sample statistic is 0.001. Bold text indicates a significant difference.

1. Macroalgal-macroalgal comparisons

| Sample | *D. menstrualis* | *G. obtusata* | *H. tuna* | *L. variegata* |
| --- | --- | --- | --- | --- |
| *G. obtusata* | **R= 0.93,**  **p = 0.048** |  |  |  |
| *H. tuna* | **R= 0.24,**  **p = 0.016** | R= 0.36,  p = 0.143 |  |  |
| *L. variegata* | **R= 0.28,**  **p = 0.024** | **R= 0.64,**  **p = 0.048** | **R= 0.25,**  **p = 0.032** |  |
| *S. polyceratium* | **R= 1.00,**  **p = 0.008** | **R= 1.00,**  **p = 0.048** | **R= 0.98,**  **p = 0.008** | **R= 1.00,**  **p = 0.008** |

1. Macroalgal-Coral comparisons

| Sample | *D. menstrualis* | *G. obtusata* | *H. tuna* | *L. variegata* | *S. polyceratium* |
| --- | --- | --- | --- | --- | --- |
| Control  Corals | **R= 0.93,**  **p = 0.018** | R= 1.00,  p = 0.100 | **R= 0.67,**  **p = 0.018** | **R= 0.98,**  **p = 0.018** | **R= 1.00,**  **p = 0.018** |
| *D. menstrualis -*Corals | **R= 0.63,**  **p = 0.008** | R= 0.64,  p = 0.133 | **R= 0.71,**  **p = 0.008** | **R= 0.72,**  **p = 0.008** | **R= 0.73,**  **p = 0.008** |
| *G. obtusata -*Corals | **R= 0.78,**  **p = 0.008** | R= 1.00,  p = 0.067 | **R= 0.77,**  **p = 0.008** | **R= 0.94,**  **p = 0.008** | **R= 1.00,**  **p = 0.008** |
| *H. tuna* -  Corals | **R= 0.97,**  **p = 0.018** | R= 1.00,  p = 0.100 | **R= 0.86,**  **p = 0.018** | **R= 1.00,**  **p = 0.018** | **R= 1.00,**  **p = 0.018** |
| *L. variegata*-Corals | **R= 0.73,**  **p = 0.008** | R= 1.00,  p = 0.067 | **R= 0.74,**  **p = 0.008** | **R= 0.85,**  **p = 0.008** | **R= 1.00,**  **p = 0.008** |
| *S. polyceratium* -Corals | **R= 0.63,**  **p = 0.008** | R= 0.79,  p = 0.067 | **R= 0.31,**  **p = 0.048** | **R= 0.85,**  **p = 0.008** | **R= 1.00,**  **p = 0.008** |

1. Coral-Coral comparisons

| Sample | *D. menstrualis -*Corals | *G. obtusata -*Corals | *H. tuna -*Corals | *L. variegata-*Corals | *S. polyceratium -*Corals |
| --- | --- | --- | --- | --- | --- |
| Control Corals | R= -0.07,  p = 0.657 | **R= 0.69,**  **p = 0.029** | R= 1.00,  p = 0.100 | R= 0.32,  p = 0.143 | **R= 0.82,**  **p = 0.029** |
| *D. menstrualis -* Corals | | R= 0.18,  p = 0.171 | R= 0.17,  p = 0.229 | R= 0.14,  p = 0.257 | **R= 0.48,**  **p = 0.029** |
| *G. obtusata -* Corals | | | R= 0.52,  p = 0.057 | R= 0.18,  p = 0.229 | **R= 0.89,**  **p = 0.029** |
| *H. tuna* - Corals | | | | R= 0.43,  p = 0.086 | **R= 0.87,**  **p = 0.029** |
| *L. variegata*- Corals | | | | | **R= 0.74,**  **p = 0.029** |
