# Supplementary material for: Macroalgae Decrease Growth and Alter Microbial Community Structure of the Reef-Building Coral, Porites astreoides
Source: PLoS One. 2012 Sep 5;7(9):e44246. doi: 10.1371/journal.pone.0044246 (PMC3434190; doi:10.1371/journal.pone.0044246)
Supplement: Table S2 — SIMPER Analysis of Macroalgae-associated Microbial Communities. Bold indicated total percent similarity. The most similar (Sim) or dissimilar (Diss) TRFs are followed by their average contribution to similarity or dissimilarity between two the macroalgae taxa. (DOCX) [file pone.0044246.s004.docx]

| Sample | *D. menstrualis* | *G. obtusata* | *H. tuna* | *L. variegata* | *S. polyceratium* |
| --- | --- | --- | --- | --- | --- |
| *D. menstrualis* | **Σ Sim=46.1%**   \| 63 \| *10.0 %* \| \| --- \| --- \| \| 51 \| *7.7 %* \| \| 54 \| *4.5 %* \| \| 1045 \| *4.0 %* \| \| 511 \| *3.7 %* \| | **Σ Diss=80.4%**   \| 51 \| *7.9 %* \| \| --- \| --- \| \| 54 \| *6.3 %* \| \| 1045 \| *5.9 %* \| \| 511 \| *5.2 %* \| \| 513 \| *4.8 %* \| | **Σ Diss=66.0%**   \| 841 \| *2.6 %* \| \| --- \| --- \| \| 826 \| *2.6 %* \| \| 825 \| *2.5 %* \| \| 1045 \| *2.5 %* \| \| 1047 \| *2.3 %* \| | **Σ Diss=66.3%**   \| 1045 \| *2.4 %* \| \| --- \| --- \| \| 513 \| *2.2 %* \| \| 511 \| *2.1 %* \| \| 424 \| *2.1 %* \| \| 510 \| *2.0 %* \| | **Σ Diss=89.7%**   \| 895 \| *1.3 %* \| \| --- \| --- \| \| 380 \| *1.3 %* \| \| 877 \| *1.2 %* \| \| 579 \| *1.2 %* \| \| 435 \| *1.1 %* \| |
| *G. obtusata* | | **Σ Sim=67.8 %**   \| 63 \| *67.8 %* \| \| --- \| --- \| | **Σ Diss=76.5%**   \| 54 \| *7.8 %* \| \| --- \| --- \| \| 1047 \| *4.8 %* \| \| 1046 \| *4.4 %* \| \| 51 \| *4.4 %* \| \| 841 \| *4.4 %* \| | **Σ Diss=81.9%**   \| 51 \| *6.6 %* \| \| --- \| --- \| \| 826 \| *4.1 %* \| \| 419 \| *3.9 %* \| \| 54 \| *3.8 %* \| \| 424 \| *3.7 %* \| | **Σ Diss=97.9%**   \| 895 \| *1.4 %* \| \| --- \| --- \| \| 380 \| *1.4 %* \| \| 877 \| *1.3 %* \| \| 419 \| *1.3 %* \| \| 579 \| *1.3 %* \| |
| *H. tuna* | | | **Σ Sim=37.4 %**   \| 63 \| *10.0 %* \| \| --- \| --- \| \| 54 \| *7.3 %* \| \| 825 \| *2.9 %* \| \| 51 \| *2.7 %* \| \| 841 \| *2.7 %* \| | **Σ Diss=71.2%**   \| 826 \| *2.5 %* \| \| --- \| --- \| \| 841 \| *2.2 %* \| \| 424 \| *2.2 %* \| \| 510 \| *2.0 %* \| \| 1045 \| *2.0 %* \| | **Σ Diss= 91.1%**   \| 895 \| *1.3 %* \| \| --- \| --- \| \| 380 \| *1.3 %* \| \| 877 \| *1.2 %* \| \| 579 \| *1.2 %* \| \| 435 \| *1.1 %* \| |
| *L. variegata* | | | | **Σ Sim=32.6%**   \| 63 \| *7.6 %* \| \| --- \| --- \| \| 51 \| *6.0 %* \| \| 826 \| *2.8 %* \| \| 54 \| *2.7 %* \| \| 419 \| *2.6 %* \| | **Σ Diss=90.0%**   \| 895 \| *1.2 %* \| \| --- \| --- \| \| 380 \| *1.2 %* \| \| 579 \| *1.1 %* \| \| 435 \| *1.1 %* \| \| 877 \| *1.1 %* \| |
| *S. polyceratium* | | | | | **Σ Sim=56.6%**   \| 895 \| *1.3 %* \| \| --- \| --- \| \| 380 \| *1.3 %* \| \| 877 \| *1.3 %* \| \| 419 \| *1.2 %* \| \| 579 \| *1.2 %* \| |
