# Supplementary material for: Macroalgae Decrease Growth and Alter Microbial Community Structure of the Reef-Building Coral, Porites astreoides
Source: PLoS One. 2012 Sep 5;7(9):e44246. doi: 10.1371/journal.pone.0044246 (PMC3434190; doi:10.1371/journal.pone.0044246)
Supplement: Table S3 — SIMPER Analysis of Coral-associated Communities After Prolonged Contact with Macroalgae. Bold indicated total similarity. The most similar (Sim) or dissimilar (Diss) TRFs are followed by their percent contribution to the total similarity or dissimilarity. (DOCX) [file pone.0044246.s005.docx]

| Sample | Control  Corals | *D. menstrualis -*Corals | *G. obtusata –*Corals | *H. tuna* –Corals | *L. variegata-* Corals | *S. polyceratium-* Corals |
| --- | --- | --- | --- | --- | --- | --- |
| Control Corals | **Σ Sim=44.3%**   \| 341 \| ***7.0*** % \| \| --- \| --- \| \| 63 \| ***6.5*** % \| \| 76 \| ***6.0*** % \| \| 670 \| ***5.2*** % \| \| 340 \| ***5.2*** % \| | **Σ Diss=76.1%**   \| 670 \| *2.3 %* \| \| --- \| --- \| \| 340 \| *2.1 %* \| \| 803 \| *1.9 %* \| \| 60 \| *1.7 %* \| \| 1048 \| *1.5 %* \| | **Σ Diss=70.5%**   \| 828 \| *2.4 %* \| \| --- \| --- \| \| 427 \| *2.3 %* \| \| 126 \| *2.3 %* \| \| 826 \| *2.1 %* \| \| 470 \| *2.0 %* \| | **Σ Diss=79.3%**   \| 826 \| *1.7 %* \| \| --- \| --- \| \| 427 \| *1.7 %* \| \| 828 \| *1.6 %* \| \| 802 \| *1.6 %* \| \| 341 \| *1.5 %* \| | **Σ Sim=70.8%**   \| 126 \| *2.9 %* \| \| --- \| --- \| \| 470 \| *2.8 %* \| \| 340 \| *2.7 %* \| \| 670 \| *2.6 %* \| \| 423 \| *2.4 %* \| | **Σ Diss=77.4%**   \| 879 \| *3.6 %* \| \| --- \| --- \| \| 564 \| *3.5 %* \| \| 671 \| *3.3 %* \| \| 341 \| *3.2 %* \| \| 670 \| *2.9 %* \| |
| *D. menstrualis –*Corals | | **Σ Sim=16.0%**   \| 76 \| *3.5 %* \| \| --- \| --- \| \| 341 \| *3.2 %* \| \| 63 \| *3.1 %* \| \| 803 \| *1.8 %* \| \| 60 \| *0.5 %* \| | **Σ Diss=76.3%**   \| 126 \| *1.9 %* \| \| --- \| --- \| \| 427 \| *1.7 %* \| \| 826 \| *1.6 %* \| \| 828 \| *1.5 %* \| \| 470 \| *1.5 %* \| | **Σ Diss=79.3%**   \| 802 \| *1.4 %* \| \| --- \| --- \| \| 126 \| *1.3 %* \| \| 826 \| *1.2 %* \| \| 899 \| *1.2 %* \| \| 427 \| *1.2 %* \| | **Σ Sim=79.7%**   \| 126 \| *2.4 %* \| \| --- \| --- \| \| 470 \| *2.0 %* \| \| 421 \| *1.8 %* \| \| 803 \| *1.8 %* \| \| 60 \| *1.6 %* \| | **Σ Diss=85.2%**   \| 564 \| *2.9 %* \| \| --- \| --- \| \| 879 \| *2.9 %* \| \| 671 \| *2.8 %* \| \| 803 \| *2.0 %* \| \| 1047 \| *2.0 %* \| |
| *G. obtusata -*Corals | | | **Σ Sim=42.7 %**   \| 63 \| *4.1 %* \| \| --- \| --- \| \| 76 \| *3.8 %* \| \| 828 \| *3.4 %* \| \| 126 \| *3.4 %* \| \| 427 \| *3.2 %* \| | **Σ Diss=65.8%**   \| 802 \| *1.4 %* \| \| --- \| --- \| \| 227 \| *1.2 %* \| \| 858 \| *1.1 %* \| \| 75 \| *1.1 %* \| \| 883 \| *1.1 %* \| | **Σ Sim=66.8%**   \| 828 \| 2.3 % \| \| --- \| --- \| \| 883 \| 1.9 % \| \| 199 \| 1.8 % \| \| 423 \| 1.7 % \| \| 826 \| 1.5 % \| | **Σ Diss=81.0%**   \| 879 \| *2.9 %* \| \| --- \| --- \| \| 564 \| *2.8 %* \| \| 671 \| *2.5 %* \| \| 427 \| *2.4 %* \| \| 828 \| *2.1 %* \| |
| *H. tuna* –Corals | | | | **Σ Sim=36.9%**   \| 423 \| *2.1 %* \| \| --- \| --- \| \| 826 \| *2.1 %* \| \| 63 \| *2.1 %* \| \| 828 \| *2.0 %* \| \| 427 \| *1.8 %* \| | **Σ Sim=75.5%**   \| 423 \| *1. 8 %* \| \| --- \| --- \| \| 828 \| *1.6 %* \| \| 882 \| *1.5 %* \| \| 839 \| *1.4 %* \| \| 899 \| *1.4 %* \| | **Σ Diss=83.8%**   \| 564 \| *1.9 %* \| \| --- \| --- \| \| 879 \| *1.8 %* \| \| 427 \| *1.7 %* \| \| 802 \| *1.6 %* \| \| 882 \| *1.5 %* \| |
| *L. variegata-* Corals | | | | | **Σ Sim=38.7%**   \| 63 \| *6.0 %* \| \| --- \| --- \| \| 421 \| *5.8 %* \| \| 76 \| *5.7 %* \| \| 126 \| *5.2 %* \| \| 470 \| *4.6 %* \| | **Σ Diss=79.1%**   \| 879 \| *3.5 %* \| \| --- \| --- \| \| 564 \| *3.4 %* \| \| 421 \| *3.3 %* \| \| 671 \| *3.2 %* \| \| 470 \| *2.9 %* \| |
| *S. polyceratium-* Corals | | | | | | **Σ Sim= 33.5**   \| 63 \| *8.7 %* \| \| --- \| --- \| \| 671 \| *6.3 %* \| \| 879 \| *4.3 %* \| \| 564 \| *4.1 %* \| \| 1047 \| *3.5 %* \| |
