# Supplementary material for: Macroalgae Decrease Growth and Alter Microbial Community Structure of the Reef-Building Coral, Porites astreoides
Source: PLoS One. 2012 Sep 5;7(9):e44246. doi: 10.1371/journal.pone.0044246 (PMC3434190; doi:10.1371/journal.pone.0044246)
Supplement: Table S4 — TRF ANOVA Data for Coral-Algal Competition Experiments – Statistical values for one way ANOVA on TRF abundance data. C = control corals, D = D. menstrualis exposed corals, G = G. obtusata exposed corals, H = H. tuna exposed corals, L = L. variegata exposed corals, and S = S. polyceratium exposed corals. (DOCX) [file pone.0044246.s006.docx]

| TRF | Pairwise differences detected | Transformation | df error | df factor | f ratio | p value |
| --- | --- | --- | --- | --- | --- | --- |
| 63 | C-S, D-S, G-S, H-S, L-S | sqrt | 15 | 5 | 6.66 | 0.002 |
| 75 | C-H, G-H |  | 15 | 5 | 3.74 | 0.021 |
| 126 | C-G, C-L, D-L, D-G, S-L, |  | 15 | 5 | 6.71 | 0.002 |
| 227 | C-H, D-H, G-H, L-H, S-H | sqrt | 15 | 5 | 34.50 | < 0.001 |
| 312 | C-H, D-H, G-H, L-H |  | 15 | 5 | 5.00 | 0.007 |
| 341 | C-G, C-D, C-G, C-H, C-L, C-S |  | 15 | 5 | 19.32 | < 0.001 |
| 423 | C-H, D-H, G-H, L-H, S-H |  | 15 | 5 | 5.93 | 0.003 |
| 564 | C-S, D-S, G-S, H-S, L-S |  | 15 | 5 | 5.55 | 0.004 |
| 670 | C-D, C-H, C-L, C-S | sin | 15 | 5 | 7.53 | 0.001 |
| 802 | C-H, D-H, G-H, L-H, S-H |  | 15 | 5 | 5.35 | 0.005 |
| 826 | C-H, S-H, L-H |  | 15 | 5 | 3.61 | 0.024 |
| 828 | C-G, C-H, D-H, G-L, G-S, H-L, H-S | sqrt | 15 | 5 | 14.64 | < 0.001 |
| 858 | C-H, D-H, G-H, L-H, S-H | sqrt | 15 | 5 | 53.68 | < 0.001 |
| 879 | C-S, D-S, G-S, L-S | log | 15 | 5 | 4.57 | 0.01 |
| 882 | C-H, D-H, G-H, L-H, S-H |  | 15 | 5 | 5.79 | 0.004 |
| 899 | C-H, D-H, G-H, L-H, S-H |  | 15 | 5 | 9.33 | < 0.001 |
